# Impact of the Food4Moms produce prescription program on readiness for healthy eating, fruit and vegetable intake, and food security

**DOI:** 10.1371/journal.pone.0348968

**Published:** 2026-09-02

**Authors:** Sofia Segura-Pérez, Katina Gionteris, Amber Hromi-Fiedler, Kathleen O’Connor Duffany, Elizabeth C. Rhodes, Sara Rodonis, Andrea Aleaga-Figueroa, Gilma Galdamez, Andrea Tristán Urrutia, Rafael Pérez-Escamilla

**Affiliations:** 1 Hispanic Health Council, Hartford, Connecticut, United States of America; 2 Wholesome Wave, Bridgeport, Connecticut, United States of America; 3 Department of Social and Behavioral Sciences, Yale School of Public Health, New Haven, Connecticut, United States of America; 4 Yale-Griffin CDC Prevention Research Center (PRC), New Haven and Derby, Connecticut, United States of America; 5 Hubert Department of Public Health, Rollins School of Public Health, Emory University, Atlanta, GeorgiaUnited States of America; 6 Emory Global Diabetes Research Center, Woodruff Health Sciences Center, Emory University, Atlanta, Georgia, United States of America; 7 Drexel University Dornsife School of Public Health, Philadelphia, Pennsylvania, United States of America; Universidade de Sao Paulo Faculdade de Saude Publica, BRAZIL

## Abstract

Produce prescription (PRx) programs targeting different populations and conditions have been found to be effective. However, few have focused on pregnant women. The objectives of this study were to assess the impact of the Food4Moms (F4M) PRx program on 1) healthy eating stages of change 2) intake of fruits and vegetables (i.e., produce), and 3) household food security among pregnant Latina women. F4M recruited low-income Latinas living in Greater Hartford, Connecticut that received a “produce prescription” from a Registered Dietitian based at the community-based organization (CBO) where the program was implemented. Participants were offered $100 per month for 10 months through Fresh Connect debit cards that only worked to purchase fresh produce from two food retailers or the equivalent value in fresh produce delivered at home. To be fully enrolled in F4M, participants had to complete a baseline survey and the first nutrition education interactive session. Enrolled participants were offered additional nutrition education sessions at the CBO and received text messages with nutrition tips as well as reminders to spend their remaining benefit balances towards the end of each month. A single-group pre-post study design was used to assess the impact of F4M 10 months after the card activation. No attrition bias was detected when comparing the characteristics of those completing (N = 113) vs. those not completing the endline survey (N = 41). Pre-post Wilcoxon signed-test or paired t-test analyses showed that F4M had a positive impact on healthy eating readiness (p < 0.001), the consumption of fruits (p < 0.001) and vegetables (p < 0.001), and household food security (p = 0.034). F4M is a promising community-engaged PRx program that may improve readiness for healthy eating, produce intake, and household food security. Implementation research is needed to find out how to effectively scale out and sustain programs like F4M. The study was registered in ClinicalTrials.gov (identifier: NCT05907616).

## Introduction

A higher intake of fruits and vegetables is consistently associated with a reduced risk of chronic diseases as well as lower all-cause mortality [1]. Despite these well-known benefits, intake levels in the United States remain suboptimal. According to the Center for Disease Control and Prevention, only 12.3% of adults meet the recommended fruit intake and 10% meet vegetable intake recommendations, with even lower consumption among individuals living in poverty (6.8%) [2]. Among pregnant women, fruit and vegetable intake is similarly inadequate. Data from the National Health and Nutrition Examination Survey (NHANES) indicate that only 35.4% meet recommended intake levels. On average, pregnant women consume 1.3 cups of fruits and 1.6 cups of vegetables per day [3], which falls short of the 2 to 2.5 cups of fruits and 2.5 to 3 cups of vegetables daily intake recommended by the Dietary Guidelines of Americans 2020–2025 [4]. Optimal nutrition before and during pregnancy is essential for maternal and fetal health. Adequate consumption of fruits and vegetables, rich in fiber, vitamin, minerals and antioxidants, has been associated with increased gut microbiota diversity, as well as a reduced risk of gestational diabetes, preeclampsia, and constipation problems [5–7]. Higher intake of fruits and vegetables has also been linked to improved self-management of gestational diabetes [8]. Maternal fruit and vegetable intake during pregnancy may further influence infant health outcomes as it may influence the infant gut microbiome at 2 months of age [9] and is positively associated with fetal and infant growth through six months [10]. At the same time, food security defined at the World Food Summit, as a condition in which “all people at all times have physical and economic access to sufficient, safe and nutritious food” [11], the lack of food security known as food insecurity remains a significant public health challenge. In 2024 13.7% of U.S. households were food insecure, with substantially higher prevalence among households below the poverty threshold (39.4%), single female headed households (36.8%), Black (24.4%), and Hispanic households (20.2%), and households with children (18.4%) [12], and between 10 and 14% of pregnant women in the US experience food insecurity [13,14], which is associated with poorer diet quality and increased risk of adverse maternal and pregnancy outcomes [15,16]. Furthermore, food insecurity is associated with limited access to nutritious and healthy foods including fruits and vegetables. More specifically, pregnant women experiencing food insecurity consume fewer vegetables [17]. A study conducted with low-income pregnant women in an urban county in the Southeast region of the USA found that 43% reported low to very low food security and those with very low food security consumed a significantly lower variety of fruits and vegetables, likely because of reduced availability of fresh produce at home [18]. These findings are consistent with other studies linking food insecurity to poorer overall diet quality and fruit and vegetable intake among pregnant women [19]. Together, suboptimal fruit and vegetable intake and food insecurity during pregnancy represent critical, interrelated public health concerns with implications for both maternal and intergenerational health.

Food assistance programs for pregnant women such as the Special Supplemental Nutrition Program for Women, Infants, and Children (WIC) improve perinatal outcomes, birth outcomes, access to nutritious foods, and prenatal care attendance [20]. Emerging evidence suggests that Produce prescription (PRx) programs are a complementary innovative strategy to improve access to healthy foods. PRx programs are based on interventions in which healthcare providers prescribe fruits and vegetables to patients, typically providing financial incentives or vouchers redeemable for fresh produce. PRx programs are likely to improve dietary behaviors, food security, and health outcomes. A systematic review of 20 PRx interventions conducted in the U.S. found positive impacts of PRx interventions on food security and half of the studies also reported significant improvements in fruit and vegetable intake, along with improvements in blood glucose control (proxied by HbA1c) among individuals with type 2 diabetes [21]. PRx programs have also been found to increase fruit and vegetable consumption among low-income participants with poor cardiometabolic health when incentives are provided for at least six months [22]. Produce prescription programs that combine fruit and vegetable incentives with nutrition education have been shown to increase fruit and vegetable intake and food security [22]. Further evidence from the evaluation of the Vouchers 4 Veggies PRx program in San Francisco, California, demonstrated that monthly incentives of $20-$40 over a period of six months significantly improved fruit and vegetables consumption and food security in a diverse, low-income population [23]. A PRx program for pregnant African American women in Flint, Michigan, provided $15.00 fruit and vegetable prescription at each prenatal visit, redeemable at farmer markets and food hubs. The program significantly improved food security by late pregnancy and into the postpartum period, while fruit and vegetable intake increased during early to mid-pregnancy but declined after childbirth [24].

PRx programs targeting different populations and conditions have been found to be highly cost-effective [25].

While these findings underscore the potential of PRx programs to improve dietary intake, food security, and health outcomes, most studies have focused on individuals with chronic diseases and very few, have focused on low-income pregnant women despite of the promising nutritional and health benefits for this population. In addition, it is important to evaluate the influence of PRx programs on behavior change mediators, such as readiness to adopt and sustain healthy eating practices.

This paper is very timely since Congress recently passed the Consolidated Appropriations Act of 2026 through the Department of Health and Human Services, providing $15 million in grants for produce prescription for pregnant women [26]

### Objectives

The objectives of this study were to assess the impact of the Food4Moms (F4M) PRx program on 1) healthy eating stages of change, 2) intake of fresh fruits and vegetables, and 3) household food security among pregnant Latina women.

## Materials and methods

### Study setting

The City of Hartford, Connecticut, has a population of 119,669 residents, of whom 44.8% identify as Hispanic or Latino; approximately 74% of this group is of Puerto Rican origin [27]. Non-Hispanic Black and non-Hispanic White residents comprise 36.1% and 15.9% of the population, respectively, and 18% of residents are younger than 18 years [28]. Hartford faces marked socioeconomic inequities. The median household income is $45,300, substantially lower than the Connecticut state median of $93,760, and 25.5% of residents live below the federal poverty line, more than twice the statewide rate (10.3%). Poverty disproportionately affects Hispanic/Latina female-headed households with children under 18 years, among whom 58% live below the poverty line, followed by Puerto Rican households (54%) and the overall Hartford population (44%) [29].

Food insecurity and financial strain are highly prevalent. In 2023, 32% of Hartford households were food insecure, 54% reported just getting by or struggling financially, and 37% of children lived in poverty [30]. Hartford is also characterized as a “food swamp,” defined by a higher density of unhealthy relative to healthy food outlets [31]. This food environment is of public health concern given evidence linking participation in food assistance programs with poorer dietary quality and increased obesity risk [32]. Collectively, these structural and social conditions indicate that Hartford is a high-priority setting for Food is Medicine (FIM) interventions.

### Study partners and codesign

This study was a collaboration between Wholesome Wave (a national non-profit organization dedicated to ending food and nutrition insecurity in the U.S.), the Hispanic Health Council, the Yale Griffin Prevention Research Center, and the Yale School of Public Health.

F4M was codesigned and tested for feasibility using community-engaged implementation science methods, the codesign phase included iterative refinements based on input through three consecutive listening sessions with 21 Latina women with low incomes who were either pregnant or had a child younger than two years and were residing in Greater Hartford, Connecticut. Following the codesign phase, the program was tested for feasibility with 20 participants. During this phase we identified implementation challenges and evaluated participant satisfaction levels. More details on the codesign and feasibility testing of the program has been published elsewhere [33]. Findings from the feasibility phase led to the addition of more content on pregnancy related topics and infant feeding to the interactive nutrition education sessions. A text message was also added with the remaining benefit balance on the card close to the end of the month. Participants in the feasibility phase reported high satisfaction with all program components [33].

This paper describes the impact evaluation of F4M, program’s participants were recruited through a community-based organization, the Hispanic Health Council Maternal Health Programs, and the local Supplemental Nutrition Program for Women, Infants and Children (WIC) Office.

F4M's impact was assessed following a single-group pre-post study design with 154 participants, which included those enrolled during feasibility phase. Throughout program design, implementation, and evaluation of F4M, community-engaged methods were applied through a person-centered lens to ensure cultural relevance, address structural barriers to healthy food access, and center the perspectives of participants. F4M was co-designed through listening sessions with pregnant and postpartum Latinas living in Hartford CT [33]. Through this approach we were able to understand women’s needs and preferences, and then iteratively design a person-centered program that is responsive to their social and cultural preferences, including language and literacy level of materials used, culturally sensitive nutrition education and the strong desire from participants to learn from each other’s food cultures given that they represented a large number of different countries of origin in Latin America and the Caribbean. Additionally, the findings from the listening sessions allowed us to design a program that considered the proximity or availability of public transportation to get to the supermarkets or food retail stores where the Fresh Connect card could be redeemed to purchase fresh produce, only. It is important to emphasize that all nutrition education personnel were highly experienced Latina community nutrition educators with decades of experience working in the community with the Hispanic Health Council SNAP-Ed program (GG, AA), under the supervision by a senior Registered Dietitian who was also Latina (SSP).

### Conceptual frameworks

**Fig 1:**
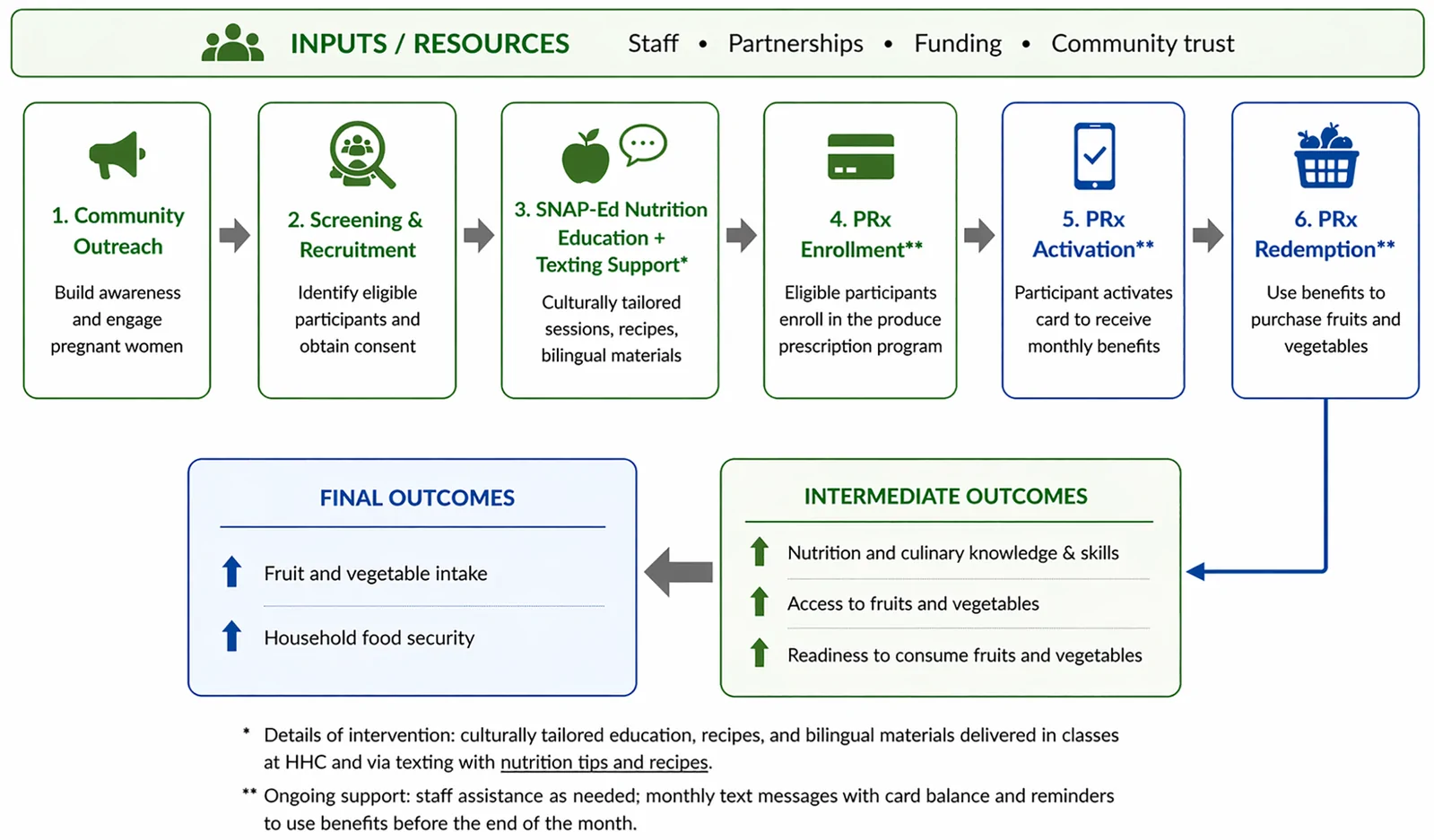
Program Impact Pathway (PIP) of the Food4Moms PRx program implemented in Hartford, Connecticut. The PIP illustrates the sequence of core program activities, from community outreach through incentive redemption, and their relationship to intermediate, and final outcomes, including improvements in fruit and vegetable consumption readiness and intake, and in household food security. Supportive actions through staff and text messages identified during the codesign and feasibility phases were integrated across all stages to enhance participant engagement and reduce barriers to program participation.

Two complementary frameworks guided program development, implementation, and evaluation across these three study phases. The Program Impact Pathway (PIP) framework was used to guide the codesign phase as well as the program implementation and evaluation phases [34]. The initial basic PIP framework for the F4M program outlined the main activities required during the study period to achieve the desired objectives and outcomes of improving produce intake and food security among study participants. These activities included: 1) community outreach; 2) screening and recruitment; 3) attendance to interactive nutrition education sessions provided by the Hispanic Health Council’s SNAP-Ed program; 4) prescription for and enrollment in the PRx program. The prescription consisted of a card stating the participant’s full name, date prescribed, and signed by the bicultural and bilingual Registered Dietitian based at the Hispanic Health Council (SSP). The prescription cards stated, “$100 a month to buy fresh fruits and vegetables only for 10 months” in either English or Spanish based on participant's language preference. The “prescription” was provided to participants once they completed the baseline survey and the mandatory nutrition session; and 5) activation and redemption of incentives that were provided as a Fresh Connect debit card for the vast majority of participants. Each of these core PIP activities were further strengthened by the addition of supportive action identified during the codesign and feasibility phase of the study, including text messages and staff support with card activation and redemption challenges. These supportive actions were intended to address barriers and facilitate participant engagement, making it easier for participants to enroll in and actively participate in the program based on their input and experiences (Fig 1).

The Capability, Opportunity, Motivation–Behavior (COM-B) model was used to further inform program design and confirm that the codesigned F4M program included the necessary components to support behavior change related to fruit and vegetable consumption (Table 1) [35].

**Table 1:** Mapping of Food4Moms Produce Prescription program components to the COM-B Framework.

COM-B Component	F4M Program Features
Capability	• Offer practical and culturally sensitive skill building interactive nutrition education (e.g., cooking demonstrations, recipe preparation, tastings) • Improved knowledge and skills to make healthier food choices and prepare fresh produce
Opportunity	• Access to a trusted community-based organization (CBO) coordinating the program • Easily accessible neighborhood supermarkets accepting electronic produce card • Alternative incentive options including home delivery • Hybrid approach to interactive nutrition education sessions (in-person + virtual) • Clear written instructions for Produce Prescription (PRx) activation and redemption, provided in participants’ preferred language • Guidance on available redemption options
Motivation	• Desire to improve baby’s and mother’s health • Financial incentive: $100 per month for 10 months • Motivational text messages with healthy eating tips, recipes, and reminders to use EBT Card balance

According to the COM-B model, behavior change occurs when individuals have the capability, opportunity, and motivation to perform a given behavior [35]. Within the F4M program, capability was strengthened through the inclusion of practical and culturally sensitive interactive nutrition education sessions designed to improve participants’ knowledge and skills related to healthy eating and food preparation. These sessions aimed to increase participants’ ability to make healthier food choices and prepare fresh produce by incorporating vegetable recipe demonstrations and recipe tasting. Opportunity was supported through multiple program features that made participation easier and more accessible. Participants have access to a trusted community-based organization (CBO) coordinating the program, easily accessible neighborhood supermarkets accepting the electronic produce card, and an alternative option of home delivery. The hybrid approach to nutrition education sessions (in-person and virtual), clear verbal and written instructions for Produce Prescription (PRx) options, enrollment, activation and redemption provided in participant’s language of preference further facilitated engagement. Motivation was addressed by different factors since they were motivated by the desire to improve their baby’s and their own health, the financial incentive of $100.00 per month for 10 months to buy fresh produce, and motivational text messages providing healthy eating tips, recipes, and reminder to fully use their monetary incentives before the end of each month.

### Human subjects

This study was submitted as a single IRB to Yale University, indicating that the Hispanic Health Council and Wholesome Wave agreed that Yale University would provide the ethical and regulatory oversight for the study. IRB approval was received from Yale University on May 10, 2023 (IRB protocol ID: 2000034840), prior to study implementation. All partners, including the Hispanic Health Council and Wholesome Wave, helped prepare and review all materials submitted to the Yale University IRB and accepted its determinations.

### Study design

This study employed a single-group pre-post study design to assess the impact of the F4M PRx program on produce intake, household food security, and readiness for healthy eating. Participants were assessed via surveys at baseline prior to program initiation and at endline which was within two months following completion of their ten-month participation in the study. A control group or comparison group was not included due to ethical considerations. Because the intervention provided financial incentives to purchase fresh produce to a highly food insecure population, withholding the intervention was deemed unethical since evidence demonstrates that financial incentives are effective in reducing food insecurity.

#### Eligibility criteria and recruitment.

Participants were eligible for the study if they met the following criteria: identified as Latina, age 18 years or older, in their first or second trimester of pregnancy, resided in the Greater Hartford area in Connecticut, were English or Spanish speaking and were eligible for or currently participating in any one of the following programs WIC, SNAP (the largest food assistance program led by the US government that transfers funds earmarked for food to low-income families), or Medicaid (state-based healthcare). Purposive sampling was used in the co-design and feasibility phases of the study. Participants were recruited by bilingual bicultural recruiters through the maternal and child health programs at the Hispanic Health Council and a local office of the Supplemental Nutrition Program for Women, Infants and Children (WIC). The same approach was used for recruitment during the intervention phase with the addition of other maternal and child community programs in the area. The outreach for recruitment was done through the distribution and posting of flyers and word of mouth. After a brief explanation of the study, women who expressed interest were screened for eligibility either in person at the Hispanic Health Council or by phone using an online screening form to make sure they met the eligibility criteria.

#### Participants enrollment in the study.

Eligible individuals who agreed to participate provided verbal informed consent in their preferred language (English or Spanish) prior to baseline data collection. A research staff member called the participants to consent them over the phone, participants were provided access to the form electronically before or during the call so that they could follow along as the research staff explained the study. Once consented, all participants received a hard or digital copy of the consent form.

**Fig 2:**
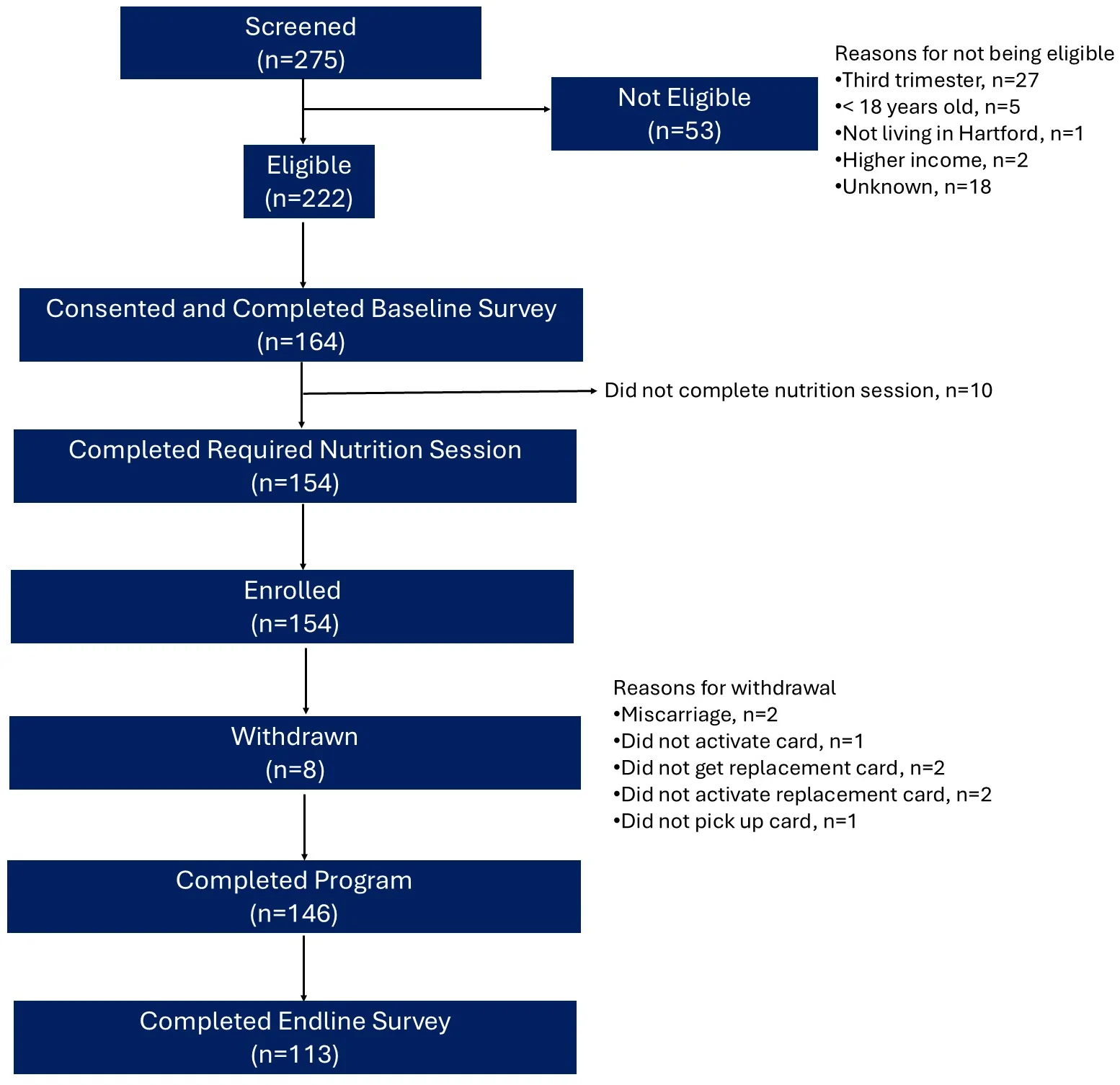
Flow chart of Food4Moms participants in program evaluation study in Greater Hartford, Connecticut.

Consented participants were sent a link by text to complete a 20-minute baseline survey in their preferred language (English or Spanish) and invited to attend the first nutrition session in the format that best suited their needs, which included in person or virtual, English/Spanish language and various times (during work hours or after work hours). Participants were considered fully enrolled in the study after providing their consent, the baseline survey, and attending the first mandatory nutrition session; all participants were also invited to the remaining nutrition education sessions offered after completing enrollment (see Fig 2).

The participant enrollment period occurred from July 24, 2023, through July 30, 2024. During this time, 275 individuals were screened, of whom 222 met the study's eligibility criteria.

A total of 164 eligible participants provided consent and completed the baseline survey (Fig 2). Of these, 10 participants who had completed the baseline survey did not attend the mandatory nutrition session and therefore did not fulfill the enrollment criteria. All participants were sent a confirmation text of the remaining nutrition sessions that they chose, and a reminder text one day before each session took place. Consequently, 154 participants were fully enrolled in the study. During the study period, eight participants were withdrawn from the study including two due to miscarriage, and six due to card related issues such as one never activated card, two never activated replacement card, two were never able to get replacement card, and one did not pick up the card at the Hispanic Health Council (Fig 2). Overall, 146 participants completed the 10-month study period, of whom 113 completed the post intervention survey. The impact analysis is based on the 113 participants that completed pre and endline surveys (Fig 2).

#### Incentives distribution options.

**Fig 3:**
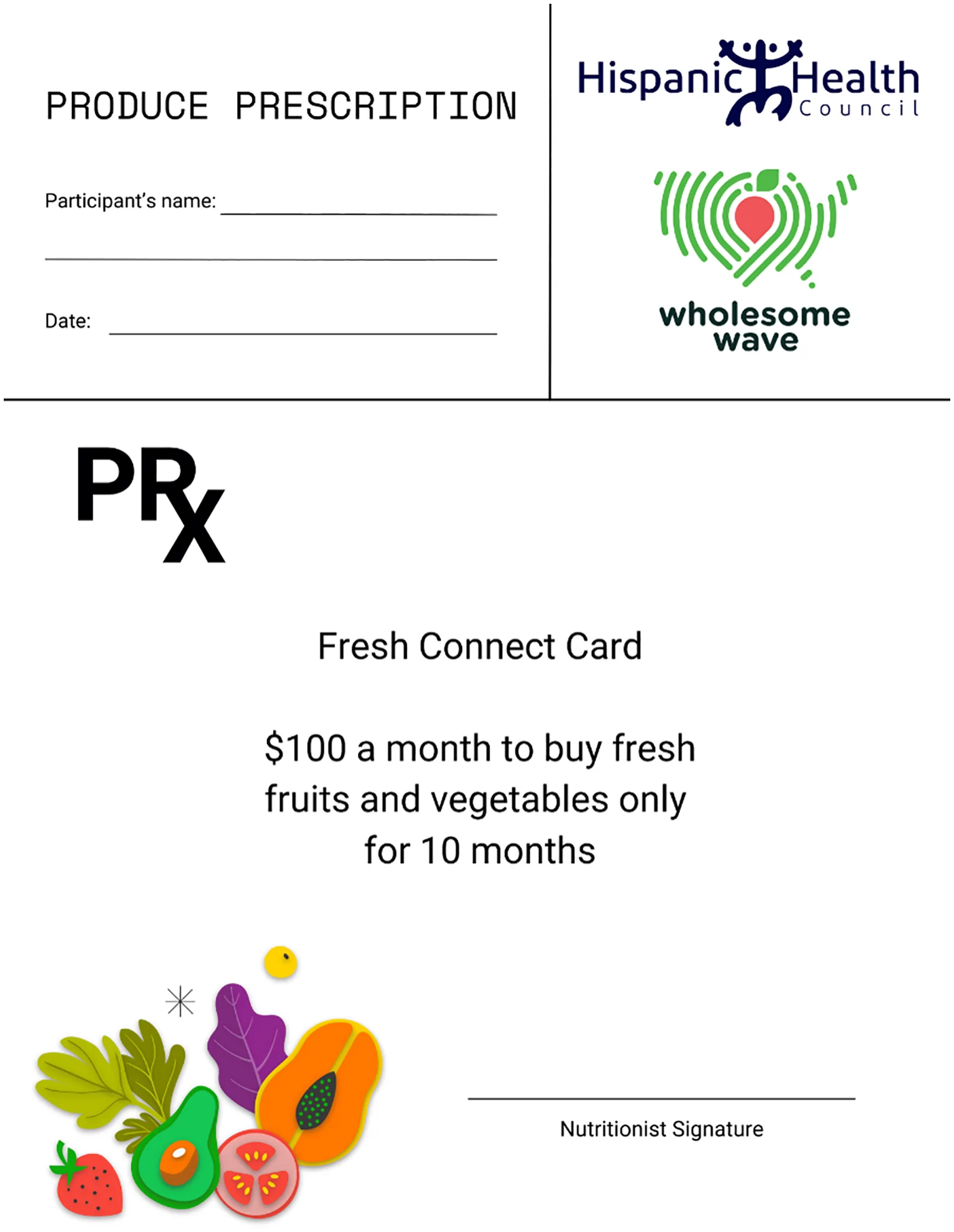
Produce “prescription” provided to Food4Moms program participants in Spanish or English in Hartford, Connecticut.

At the conclusion of the first nutrition session, research staff explained the two options available for redeeming incentives, a Fresh Connect debit card that could only be used to purchase fresh produce, and nothing else or home delivery of fresh produce, both of which are described in the incentive section below. The vast majority of them chose the Fresh Connect option and received a “produce prescription” that consisted of a paper card stating the participant’s full name, date prescription was made and signed by the bicultural and bilingual F4M Registered Dietitian based at the Hispanic Health Council (SSP). The prescription card stated, “$100 a month to buy fresh fruits and vegetables for 10 months” in either English or Spanish based on participant's language preference (Fig 3).

As indicated in Fig 3, the Fresh Connect Card was a restricted-use debit card that was earmarked for the purchase of fresh produce at two authorized retailers, Stop & Shop supermarkets and Walmart. F4M provided monthly incentives of $100 for 10 months for a total of $1000. Incentives were preloaded onto the card at the beginning of each month. Unused funds did not roll over to subsequent months, meaning that any remaining benefit balance at the end of the month was forfeited and not added to the following month’s allocation.

Participants who selected the home delivery option received fresh produce through orders placed twice a month, one at the beginning and the other at the middle of the month via Instacart (a leading online grocery marketplace company that provides same-day delivery and curbside pickup services across the US and Canada). Orders were submitted by the project director at Wholesome Wave, who selected a standard seasonal produce package with a value equivalent to the $100 monthly incentive, independent of delivery or service fees. This approach ensured that participants received an equivalent value of fresh produce regardless of the incentive redemption method selected. Participants receiving home delivery did not have the ability to choose the specific types of fresh produce included in the delivery but had the option to select an equal mix of fruits and vegetables or an order that had more vegetables or more fruit. Participants were told that they would be given the opportunity to change their selection at the 3-month mark of the study. Most participants selected the Fresh Connect debit card, but there were exceptions. We initially had three participants requesting the produce delivery box option; however, since we needed a minimum order of 20 boxes, we were unable to fulfill these selections. Those three participants were provided with the Fresh Connect card. Within a month we were able to develop a produce delivery option using Instacart.

There were also some participants who had a very difficult time receiving the card in the mail resulting in ordering multiple replacement cards. As a result, two of those participants were provided with the Instacart produce delivery option while the Fresh Connect card issues were resolved. In January of 2025, the Fresh Connect Fidelity National Information Services (FIS) payment processor changed its banking partner and at the same time Fresh Connect moved from personalized cards to anonymous cards. Therefore, all participants still enrolled in the program after January 1^st^, 2025, needed new cards mailed to them. There were multiple issues with mailing new cards to participants resulting in participants going without cards for one to two months. In the interim period, we provided Instacart to the five participants affected so that they could have access to fresh fruits and vegetables.

#### Interactive skill-building nutrition education sessions.

To be fully enrolled in the study, participants were required to complete the first nutrition education session in their preferred language (English/Spanish) and format of their preference (in-person or online). At the end of this mandatory session, participants were then enrolled to receive their “prescription” (i.e., incentives to purchase fruits and vegetables). After enrollment, participants were invited to the rest of the nutrition education sessions offered by the Hispanic Health Council SNAP-Ed program. The nutrition skill-building interactive sessions were offered at the Hispanic Health Council and synchronously via zoom for those that could not attend in person. The sessions were designed as group sessions and included healthy culturally sensitive vegetable recipes for preparation and tasting activities.

The sessions were delivered in person or virtually with morning and evening schedules, and in participant’s language of preference (English/Spanish). The interactive nutrition education curriculum was developed specifically for F4M and was informed by the 2020 Dietary Guidelines for Americans, as well as community members feedback obtained during the codesign and feasibility phases. In addition, culturally sensitive sessions were integrated throughout the curriculum to reflect participants’ cultural food practices, beliefs, and preferences, including discussions addressing cultural perceptions of pregnancy and postpartum nutrition. All sessions included a vegetable-based recipe demonstration and lasted approximately 90 minutes. As previously mentioned, after completion of the initial feasibility phase, an additional fifth session focused on infant feeding recommendations during the first year of life was incorporated into the curriculum. Because the skills-based interactive nutrition education sessions were delivered through the federally funded SNAP-Ed program the nutrition recommendations by law had to adhere to the Dietary Guidelines for Americans. However, the specific food and recipe examples used, and the modes of education delivery were culturally tailored, considering the cost and seasonal availability of different types of fresh produce, to meet the preferences of the participants, in other words, they were person-centered.

The nutrition education sessions focused on Healthy Eating during Pregnancy (mandatory); Dietary strategies for the prevention and management of pregnancy complications; Making Healthy Food Choices Using the Nutrition Facts Label; Food Safety and Practical Nutrition Tips; and Maternal Nutrition and Infant Feeding during the first year of lif**e** (Table 2).

**Table 2:** Content of Food4Moms bilingual and interactive nutrition education sessions delivered by community nutritionists to pregnant Latinas in Hartford, Connecticut.

Module	Content Description	Activities
Healthy Eating during Pregnancy	Overview of prenatal nutrition and its impact during the first 1,000 days. My Plate, caloric needs, recommended portions, healthy snacks, and physical activity.	Building your own healthy plate; vegetable recipe preparation and tasting. Highlighting vegetables preference from different Latin American countries.
Dietary Strategies for the Prevention and Management of Pregnancy Complications	Dietary management of common pregnancy symptoms (nausea, food aversions, constipation, heartburns). Dietary recommendations for the prevention of pregnancy complications (anemia, gestational diabetes).	Interactive discussions on pregnancy food aversions, cultural beliefs on nutrition and pregnancy complications. Healthy snacks tips respecting cultural preferences.
Making Healthy Food Choices using the Food Label Nutrition Facts	Understanding the Nutrition Facts Label: serving size, calories, nutrients, ingredients, % Daily Value. The NOVA classification to identify ultra-processed foods.	Compare and interpret food labels. Culturally appropriate vegetable recipe preparation and tasting.
Food Safety during Pregnancy and Practical Nutrition Tips	Food safety during pregnancy, using the 4 steps to avoid food-borne illnesses. Nutrition tips recommendations.	Interactive discussion on food safety during pregnancy. Vegetable recipe preparation and tasting.
Maternal Nutrition and Infant Feeding During the First Year of Life	Maternal nutrition during the post-partum period. Overview of infant feeding during the first year of life, Introduction of solids and responsive feeding recommendations	Interactive discussion, including breastfeeding myths and cultural beliefs. Baby food vegetable recipes and preparation.

We used several nutrition education strategies to address the large heterogeneity in terms of countries of origin in Latin America and the Caribbean through the listening sessions and the three decades of experience of the Hispanic Health Council serving this population though their SNAP-Ed program. First, we included interactive healthy vegetable recipes preparation and tasting activities that allowed participants to learn from each other’s food cultures. An example was a quinoa-based recipe that was very well liked by participants from countries such as Mexico and Central America that were not familiar with quinoa. Another example of cultural tailoring was the way we engaged participants during the sessions. We asked participants to share cultural practices, beliefs, and family advice related to food during pregnancy and breastfeeding. Based on these discussions, we addressed respectfully topics such as how to manage cravings, food aversions, and common concerns about maternal and infant nutrition during pregnancy and lactation, connecting them to evidence-based nutrition guidance.

We avoided presenting a single “Latina/Hispanic diet” by inviting participants to share the foods, recipes and preparation methods that were most common in their families and places of origin. Rather than discouraging traditional foods, we emphasized practical strategies to adapt familiar dishes, such as adding vegetables, choosing healthier cooking methods, adjusting portion sizes, and reducing added sugar, sodium or saturated fat when possible.

As part of this approach, the educational sessions incorporated practical, participant-centered activities that allowed women to connect the nutrition concepts with their own food practices. For example, after introducing the main food groups, participants were invited to identify and add the foods they most consumed within each category. Using visual materials with a wide variety of culturally familiar foods, we asked participants to practice planning breakfasts, lunches, dinners, and snacks that reflected their usual dietary patterns and food preferences. This strategy allowed us to deliver interactive nutrition education in a way that felt familiar, accessible, and culturally meaningful to participants.

A second strategy was to allow the co-design and feasibility phase participants to contribute to curriculum design. For example, a whole module was added on specific pregnancy nutrition issues such as how to handle aversions to healthy foods, morning sickness, and another section on breastfeeding and complementary feeding. Likewise, a whole module on food safety during pregnancy was added in response to feedback from the co-design and main study participants.

Throughout the program, all participants received weekly text messages with nutrition tips, links to recipes, plus monthly reminders to use their produce incentives.

### Data collection

#### Survey instruments.

A baseline survey and an endline survey were administered through an electronic link sent to participants via text. The surveys included pre-validated scales in English and Spanish, and those pertaining to this paper are described in the data collection section. Participant’s sociodemographic data at baseline (n = 154) and among those who completed both pre and posttest (n = 113) included: participant’s age, spoken language, household size, educational attainment, monthly income, employment status, number of children in household, country of origin, ethnicity, race. We collected data on participation during the last 12 months in the following government food assistance and health coverage programs: (1) the Special Supplemental Program for Women, Infants, and Children (WIC), (2) the Supplemental Nutrition Assistance Program (SNAP); and (3) Medicaid.

#### Baseline survey.

The baseline survey took approximately 30–45 minutes to complete. Participants could complete the survey independently online (77%), have it administered by a trained bilingual and bicultural interviewer via phone or in person (9.1%) or in person but reading the questions to themselves (9.7%). In addition to collecting socio-demographic and food assistance information, the survey asked about barriers to fruits and vegetables consumption; maternal physical health and pregnancy-related complications; main sources of nutrition knowledge during pregnancy; planned infant feeding practice; state of change for healthy eating, self-efficacy related to fruit and vegetable consumption and eating habits; a food frequency questionnaire focusing on fruit and vegetable consumption; household food security.

#### Endline survey.

The endline survey had similar duration as the baseline survey, and included all measures from the baseline survey, along with additional postpartum questions related to maternal and infant health, pregnancy outcomes, infant feeding practices, and participant satisfaction with the program. The survey was intended to be completed within 2 months of program completion. Approximately 80% of the participants completed the endline survey within this timeframe, while the remaining 20% took longer to complete it.

### Data variables

#### Produce intake.

Fruit and vegetable intake were assessed at baseline and endline by using 9 items from the Dietary Screener Questionnaire assessment tool developed by the National Cancer Institute [36], that were pertinent for the assessment of fruit and vegetable consumption which was one of the primary outcomes of the study. The questions mainly probed for fruits and vegetables that were not coming from ultra-processed foods, but it is still possible that some of them were. Participants were asked how often they consumed fruits and vegetables over the past month, with responses converted to cups/day. The specific questions asked were:

Fruit intake questions

1 During the past month, how often did you eat FRUIT like bananas, oranges, melon, or any other fruit? INCLUDE fresh, frozen, canned, or dried fruit. DO NOT INCLUDE juices.
2 During the past month, how often did you drink 100% PURE FRUIT JUICES such as orange, apple, grape, etc.? DO NOT INCLUDE fruit-flavored drinks with added sugars like Capri-Sun, Sunny D, or other fruit-flavored drinks.

Vegetable intake questions

1. GREEN LEAFY OR LETTUCE SALAD, with or without other vegetables?
2. During the past month, how often did you eat any kind of FRIED POTATOES like French fries, tater tots, hash brown potatoes, or other fried potatoes?
3. During the past month, how often did you eat OTHER KIND OF POTATOES that aren’t fried, like baked, boiled, mashed, or potatoes used in soups or stews?
4. During the past month, how often did you eat refried beans, baked beans, pinto beans, black beans, beans in soup, or any other type of COOKED BEANS? INCLUDE canned or dry beans. DO NOT INCLUDE green beans or string beans.
5. During the past month, how often did you eat other VEGETABLES that were not deep-fried? These are vegetables like carrots, broccoli, collards, green beans, corn, or other vegetables that are not deep-fried. INCLUDE canned, frozen, or fresh vegetables. ALSO INCLUDE vegetables that are raw, boiled, broiled, baked, grilled, stir-fried, or microwaved.
6. During the past month, how often did you eat packaged or homemade SALSA made with tomato?
7. During the past month, how often did you eat TOMATO SAUCE in recipes such as spaghetti, lasagna, or other dishes? DO NOT INCLUDE tomato sauce in pizza.

The Dietary Screener Questionnaire (DSQ) data into cups of fruits and vegetables consumed daily. The algorithm [36] first converts the data to daily frequency and then calculates age- and gender- specific portion sizes which are then converted to cups/day of intake. The following dietary intake indicators were generated for each participant: a) intake of fruits and vegetables including legumes and French fries (cup equivalent) per day, b) intake of fruits and vegetables including legumes and excluding French fries (cup equivalent per day, c) intake of fruits (cups equivalent) per day, d) intake of vegetables including legumes and French fries (cup equivalent) per day, e) intake of vegetables including legumes and excluding French fries (cup equivalent per day).

#### Stage of change for healthy eating behaviors.

Stages of changes or readiness for healthy eating were assessed with a standardized questionnaire based on the Transtheorical Model, which classifies individuals into the precontemplation, contemplation, preparation, action, or maintenance stages [37]

#### Household food insecurity.

Food security was assessed at baseline and endline using the six-item short form of the U.S. Household Food Security Survey Module (USDA ERS). An additive score was computed based on the number of items affirmed. Participants’ scores were classified into one of the following mutually exclusive four categories using the recommended cut-off points: food secure (score –0); marginal food security (score = 1), 2–4 – low food security (score 2–4) and very low food security (scores 5–6) [38–40]

#### Covariates.

Gestational age at enrollment was assessed in the baseline survey by asking participants, “How many weeks pregnant are you?” This question was added after 20 participants had already been enrolled.

Household income was assessed in the baseline survey by asking participants, “What would you say is the total amount of money your household receives per month including money from all salaries/work, government assistance, and (if applicable) unemployment?” Participants could select one of the following response options included $0 to $999; $1,000-$1,999; $2,000-$3,000; or> $3,000. Since only 4 respondents chose “More than $3,000”, they were combined with the $2,000-$3,000 category to form the new category “$2,000 or more”.

Perceived general health status was measured using a single question which did not refer to a specific period of time. This question came from the US Centers for Disease Control and Prevention – Behavioral Risk Factor Surveillance System (BRFSS) survey [41]. Participants were asked “Would you say that in general your health is poor, fair, good, very good, or excellent?”

### Statistical analyses

All data were analyzed using IBM SPSS for Windows® (V. 21) [42]. We generated socio-economic and descriptive characteristics at baseline for all the samples and compared them (N = 154) with endline surveys. We conducted an attrition bias analysis by comparing the characteristics of those completing (N = 113) vs. those not completing the endline survey (N = 41), using the Chi-Square test. We compared the stages of change for healthy eating responses and the household food insecurity classification at baseline and endline using the non-parametric Wilcoxon signed-rank test. We compared the proportion affirming at least one item of the Household Food Security scale at both time points with the Wilcoxon test. The cup equivalents of vegetable and fruit intake were compared at baseline and endline using the paired t-test. Statistical significance was set at a p-value <=0.05. Marginal significance was considered at a p value >0.05–0.10. Multivariable analyses were not necessary because there was no evidence of attrition bias among participants that completed the endline survey compared with those that didn’t. Furthermore, this decision was supported by the fact that we did not find any significant associations between produce intake and covariates that could theoretically confound the findings including gestational age, WIC and SNAP participation, baseline food security, income, and educational level (data not shown).

## Results

### Sample characteristics

As shown in Table 3, 64.9% of the 154 enrolled participants chose to complete the baseline survey in Spanish, and a similar proportion (59.7%) were between 5 and 20 weeks of pregnancy.

**Table 3:** Baseline descriptive characteristics of Food4Moms participants, including comparisons with those that completed the endline survey.

		Baseline-All		Baseline-endline survey completers		
		N	%	N	%	P value^1^
Survey Language	English	54	35.1%	40	35.4%	.886
	Spanish	100	64.9%	73	64.6%	
	All	154	100%	113	100%	
Weeks of pregnancy at recruitment	5-12 weeks	35	22.7%	25	22.1%	.764
	13-16 weeks	32	20.8%	26	23.0%	
	17-20 weeks	25	16.2%	18	15.9%	
	21-24 weeks	26	16.9%	17	15.0%	
	25-32 weeks	16	10.4%	11	9.7%	
	Don’t Know	20	13.0%	16	14.2%	
	All	154	100%	113	100%	
Age group	18–25	55	35.7%	37	32.7%	.492
	26–30	46	29.9%	36	31.9%	
	31–40	48	31.2%	37	32.7%	
	41 or higher	5	3.2%	3	2.7%	
	All	154	100%	113	100%	
Number of pregnancies	1	44	30.1%	30	28.0%	.475
	2	44	30.1%	35	32.7%	
	3–5	58	39.7%	42	39.3%	
	All	154	100%	107	100%	
Respondent’s Birth Country/Territory	Puerto Rico	31	22.5%	13	16.5%	.054
	Peru	26	18.8%	21	20.4%	
	United States	17	12.3%	13	12.6%	
	Dominican Republic	15	10.9%	10	9.7%	
	Guatemala	10	7.2%	8	7.8%	
	Other (specify)^2^	39	28.3%	34	33.0%	
	All	138	100%	99	100%	
Respondent’s education level	Less than HS diploma or equivalent	21	14.9%	16	15.1%	.160
	High school/GED completed or some college	110	78.0%	85	80.2%	
	BS degree or higher	10	7.1%	5	4.7%	
	All	141	100%	106	100%	
Monthly income	Less than $1,000	63	42.6%	46	42.2%	.797
	$1,000 to $1,999	49	33.1%	35	32.1%	
	$2,000 or more	36	24.3%	28	25.7%	
	All	148	100%	109	100%	
Employment status	Working full time or part time	53	34.6%	36	32.1%	.713
	Unemployed/Student/Home maker	86	56.2%	66	58.9%	
	Disability/SSI/SSDI^3^	3	2.0%	2	1.8%	
	Other	11	7.2%	8	7.1%	
Access to a reliable car	All	153	100%	112	100%	
	Yes	89	57.8%	64	56.6%	.873
	No	38	24.7%	29	25.7%	
	Sometimes	27	17.5%	20	17.7%	
	All	154	100%	113	100%	
Adults living in household	1–2	98	65.8%	68	63.9%	.397
	3–5	37	24.8%	30	27.8%	
	5 or more	14	9.4%	10	9.2%	
	All	149	100%	108	100%	
Children < 18 y old living in household	None	34	23.6%	23	21.7%	.419
	1–2	85	59.0%	66	62.3%	
	3 or more	25	17.4%	17	16.0%	
	All	144	100%	106	100%	
Household Food Security Category	High food security	29	21.6%	22	22.7%	.882
	Marginal food security	18	13.4%	14	14.4%	
	Low food security	67	50.0%	47	48.5%	
	Very low food security	20	14.9%	14	14.4%	
	All	134	100%	97	100%	
Enrolled in WIC in last 12 months	No	51	33.1%	38	33.6%	.823
	Yes	103	66.9%	75	66.4%	
	All	154	100%	113	100%	
Household received SNAP benefits in past 30 days	No	104	67.5%	81	71.7%	.152
	Yes	46	29.9%	30	26.5%	
	Don’t know/Prefer not to answer	4	2.6%	2	1.8%	
	All	154	100%	113	100%	
Perceived general health status	Poor or Fair	48	31.4%	35	31.3%	.986
	Good	70	45.8%	51	45.5%	
	Very Good or Excellent	35	22.9%	26	23.2%	
	All	153	100%	112	100%	

^1^When comparing those with vs. those without completed endline surveys with Chi-square test, none of the differences were statistically significant or marginally significant.

^2^Less than 10 participants were born in each of the following countries: El Salvador (n = 8), Mexico (n = 8), Venezuela (n = 5), Colombia (n = 4), Nicaragua (n = 2), Brazil (n = 1), Ecuador (n = 1), Italy (n = 1), Panama (n = 1), There were 2 participants who misunderstood the question thinking that it referred to the place of birth of the baby.

^3^SSI: Supplemental Security Income; SSDI: Social Security Disability Insurance.

Most participants (78%) had completed high school, 87.7% were born outside of the continental United States, and only 34.6% reported being employed. Additionally, 75.7% reported a monthly income at or below $1999, and only 57.8% always had access to a car. Only 21.6% of participants experienced high food security, while 13.4% experienced marginal food security, 50% low food security, and 14.9% very low food security. Additionally, 29.9% of the participants were receiving SNAP benefits, 66.9% were enrolled in WIC, and 68.7% reported their health as good to excellent at baseline. There were no significant differences in socioeconomic characteristics between participants who completed the endline surveys and those who did not.

Of the 113 mothers who had baseline and endline data, 111 had complete information on birth outcomes and all had delivered their babies by endline. The babies age at endline ranged from 3 to 11 months with a mean of 6.5 months (SD 1.58). The 5 babies at the high end of the range (10–11 months) were in the F4M program for an extended period of time due to difficulties with the Fresh Connect cards being delivered to their home address or because their endline survey was completed 3 months past the end of the program.

### Healthy eating readiness

Between baseline and endline, participants shifted toward more advanced stages of change for healthy eating (Wilcoxon signed-rank test, p < .001) (Table 4).

**Table 4:** Stages of change for healthy eating among Food4Moms participants in Hartford, Connecticut^1^ N = 113.

	Baseline		Endline	
	N	%	N	%
I have not been eating healthy, and I do not intend on changing my eating habits in the near future.	3	2.7	3	2.7
I intend to eat healthier in the next six months.	51	45.1	20	17.7
I intend to eat healthier in the next month.	20	17.7	20	17.7
I have been eating healthier in the last six months.	32	28.3	38	33.6
I have been eating healthy for more than six months.	7	6.2	32	28.3
All	113	100%	113	100%

^1^Wilcoxon Signed ranks test, p < 0.001.

At baseline, most participants were in earlier stages with 45.1% reporting intention to eat healthier within the next six months and 17.7% within the next month, while a low percentage of participants were in the action stage (28.3%) or maintenance stage (6.2%) for eating healthy. At endline, 33.6% of participants were at the action stage for healthy eating, and 28.3% were at the maintenance stage.

### Produce intake

Among 113 participants with both a baseline and endline survey, 101 had complete data on fruit and vegetable intake. Participation in F4M was positively associated with an increase in fruit and vegetable intake (Table 5).

**Table 5:** Changes in fruit and vegetable intake among Food4Moms participants in Hartford, Connecticut.

		Baseline Cup equivalents per day		Endline Cup equivalents per day		
	N	Mean	SD	Mean	SD	Paired t-test p-value
fruits and vegetables including legumes and French fries	101	2.3043	.62226	2.5953	.71793	< 0.001
vegetables including legumes and French fries	103	1.3641	.28914	1.5462	.40313	0.001
vegetables excluding legumes and excluding French fries	103	1.2478	.29154	1.4432	.43021	< 0.001
fruits (cup equivalents) per day	108	.9829	.51476	1.1199	.55842	< 0.001

Total fruit and vegetable consumption (including legumes and French fries) increased from a mean of 2.30 to 2.59 cup equivalents per day (p < 0.001). Consumption of vegetables (including legumes and French fries) increased from 1.36 to 1.54 cup equivalents per day (p = 0.001), while vegetable intake excluding legumes and French fries increased from 1.24 to 1.44 cup equivalents per day (p < 0.001). Likewise, fruit intake increased from 0.98 to 1.11 cup equivalents per day (p < 0.001).

### Household food insecurity

Table 6 presents changes in household food insecurity scale items and for the household overall among F4M participants from baseline to endline.

**Table 6:** Household food insecurity among Food4Moms participants in Hartford, Connecticut. Assessed with the 6-item US Household Food Security Survey Module^1^.

	Never true	Sometimes true	Always true	DK
% (N)				
**Food did not last**				
baseline	29.7 (44)	45.9 (68)	19.6 (29)	4.7 (7)
endline	33.6 (38)	49.6 (56)	9.7 (11)	7.1 (8)
**Balanced meals unaffordable**				
baseline	29.5 (43)	52.7 (77)	16.4 (24)	1.4 (2)
endline	42.5 (48)	41.6 (47)	9.7 (11)	6.2 (7)
	**Yes**	**No**		**DK**
**Skipped meal** ^**2**^				
baseline	23.6 (33)	70.7 (99)		5.7 (8)
endline	22.1 (25)	73.5 (83)		4.4 (5)
**Eat less**				
baseline	29.4 (42)	67.1 (96)		3.5 (5)
endline	29.2 (33)	67.3 (76)		3.5 (4)
**Hungry**				
baseline	22.4 (33)	72.8 (107)		4.8 (7)
endline	16.8 (19)	77.9 (88)		5.3 (6)
**Household food insecurity classification**				
	**Baseline**	**Endline**	**Wilcoxon test p-value**	
	% (N)			
**4-levels HFI classification** ^**3**^			0.068	
Food secure	22.7 (22)	31.1 (33)		
Marginal FS^4^	14.4 (14)	15.1 (16)		
Low FS	48.5 (47)	37.7 (40)		
Very low FS	14.4 (14)	16 (17)		
**Ever food insecure** ^**5**^			0.034	
yes	77 (87)	67.3 (76)		

^1^6-item US Household Food Security Survey Module questions: a) The food that we bought just didn’t last, and we didn’t have money to get more; b) We couldn’t afford to eat balanced meals; c) In the last 30 days, did you or other adults in your household ever cut the size of your meals or skip meals because there wasn’t enough money for food?; d) (if yes) In the last 30 days, how many days did this happen?; e) In the last 30 days, did you ever eat less than you felt you should because there wasn’t enough money for food?; f) In the last 30 days, were you ever hungry but didn’t eat because there wasn’t enough money for food?

^2^If a participant responded yes then they were asked, in the last 30 days how many times did this happen?

^3^Household Food Insecurity classification using the recommended cut-off points.

^4^FS: Food Security.

^5^Defined as affirming any of the 6 questions asked.

The proportion of participants reporting that food did not last as “always true” decreased from 19.6% at baseline to 9.7% at endline, while those reporting this as “never true” increased from 29.7% to 33.6%. Similarly, the proportion of participants reporting that balanced meals were unaffordable as “always true” decreased from 16.4% to 9.7% with a corresponding increase in those reporting “never true” from 29.5% to 42.5%. Changes in more severe food insecurity behaviors were modest. The proportion of participants reporting skipping meals decreased slightly from 23.6% to 22.1%, and those reporting being hungry but not eating decreased from 22.4% to 16.8%, while reports of eating less remained largely unchanged.

Overall, household food security improved modestly. The proportion of participants classified as food secure increased from 22.7% to 31.1%, while those with low food security decreased from 48.5% to 37.7%, and this change was marginally significant (Wilcoxon test, p = 0.068). Furthermore, the proportion of participants experiencing any food insecurity as proxied by affirming at least one of the six scale items decreased from 77.0% to 67.3% which was statistically significant (Wilcoxon test, p = 0.034).

## Discussion

Most PRx programs are delivered through healthcare systems. In contrast, Food4Moms is a person-centered, community-based model led by a community-based organization that co-designed the program with pregnant and post-partum women with low incomes to align with their needs, preferences, and values, and delivered the program. The Food4Moms quality assurance system enabled timely identification and resolution of implementation challenges with input from all partners, including program participants. In F4M the “prescription” was provided by the agency’s Registered Dietitian as part of a SNAP-Ed Program run by the Hispanic Health Council. This approach likely helped reduce barriers to participation, such as limited access to healthcare, transportation challenges, distrust of clinical systems, while leveraging trusted community relationships to improve program reach and engagement. This approach is especially important when serving hard to reach populations, particularly recent immigrants who may not qualify for food assistance programs; notably, in this study most participants were born outside of continental United States. Participation in F4M was positively associated with improved readiness for healthy eating, and a higher intake of both fruits and vegetables. This suggests that changes in readiness to adopt healthy eating behaviors may translate into measurable dietary improvements.

The findings from our study align with the results of prior research on PRx programs that demonstrate improvements in diet quality and food security among other groups. For example, a pooled multisite evaluation of nine Wholesome Wave PRx programs across 12 U.S. states (n = 3,881), where three programs focused on 2–18 years old children and six on adults with cardiometabolic diseases, found significant increases in fruit and vegetable intake, as well as improvements in food security and self-reported health among low-income participants receiving produce incentives through vouchers or electronic cards [43]. Similarly, a Canadian healthcare-based PRx program providing weekly vouchers alongside nutrition education over 12 months to adult women and men reported reductions in food insecurity, increased fruit and vegetable intake, and improvements in select clinical biomarkers, including triglycerides, fasting insulin, and vitamin C levels [44].

Additional evidence from pediatric and family-based PRx program further supports these findings. A scoping review of 19 pediatric PRx programs providing vouchers or food boxes found a reduction of food insecurity in five out of the six studies assessing changes in food insecurity and increases in fruit and vegetable intake in five out of the seven studies measuring dietary intake [45]. Qualitative and mixed-methods studies also reported broader benefits, including reduced financial strain, improved nutrition knowledge, and enhanced cooking practices, particularly when programs included home delivery or were integrated into existing community services [46,47].

Although there is a growing body of evidence on the effectiveness of PRx programs, there is a dearth of evidence on PRx programs specifically targeting pregnant women, despite the heightened nutritional needs during pregnancy and the potential for these programs to have intergenerational health impacts. This is a critical gap addressed by our study. One notable example is a PRx program implemented among pregnant African American women in Flint, Michigan, which provided $15 fruit and vegetable prescriptions at prenatal visits. That program improved food security through pregnancy and into the postpartum period; however, increases in fruit and vegetable intake observed during pregnancy were not sustained after childbirth [24]. These findings underscore both the promise of PRx programs during pregnancy and the need for continued support in the postpartum period.

Food4Moms builds on and extends this limited evidence base by targeting a predominantly Hispanic, immigrant population and integrating culturally tailored interactive nutrition education, recipe demonstrations, and ongoing engagement through text messaging. To our knowledge, few PRx programs for pregnant women have combined financial incentives with comprehensive, culturally responsive nutrition education delivered in a community setting. This integrated approach may be particularly important for sustaining behavior change beyond pregnancy.

Our findings are consistent with a recent systematic review of Food is Medicine programs for pregnant women, which found improvements in diet quality, household food security, and some birth outcomes. Indeed, our findings support the potential benefit of PRx programs on these outcomes during this critical life stage. However, the review also highlighted substantial heterogeneity in program designs and outcomes, as well as a lack of standardized measures and long-term follow-up, limiting conclusions about overall effectiveness [22]

Our study documents that our effect size of 0.29 cup equivalents per day is in line with previous studies examining the impact of nutrition incentives studies on fruit and vegetable intake in the US [48]. Furthermore, an improvement of 0.29 cups in fruit and vegetable intake is of clinical and public health significance based on the inverse dose-response relationship between fruit and vegetable intake and mortality risk documented in the US Nurse’s Health and the Health Professionals Follow-up prospective cohort studies [49]. Furthermore, an increase of 0.29 cups per day of fruits and vegetables translates into 2 additional cups per week or 8.7 cups per month, which is a substantial amount in a population where the great majority of people do not meet the recommended intake of fruits and vegetables.

We found an inverse association between participation in Food4Moms and household food insecurity using two indicators from the same scale: any-item affirmation and level of household food insecurity. The association was statistically significant for any-item affirmation and marginally significant for the level-based indicator. Given a 37% relative increase in food security (22.7% at baseline vs. 31.1% at endline)—an effect size consistent with prior Food is Medicine intervention studies in the US [43,50] we consider this result to be meaningful, and to warrant confirmation through future studies.

Taken together, our findings contribute to a small but growing literature suggesting that PRx programs for pregnant women may improve dietary behaviors and household food security. They also highlight the potential added value of community-based, culturally tailored models in reaching underserved populations and addressing structural barriers to healthy eating.

An important limitation of this study is the use of a single-group pre–post design which limits our ability to establish causality and sort out program effects from external influences. Without a comparison group, it is difficult to disentangle intervention effects from changes naturally associated with pregnancy progression. Pregnancy itself is a period characterized by increased health awareness, greater motivation for dietary change, prenatal counseling exposure, and heightened concern for maternal and fetal health. On the other hand, pregnancy is also a period where women can experience food cravings as well as aversions. Therefore, behavioral improvements may have occurred independently of the intervention. Another potential limitation is that social desirability bias may have been present in the study, given that participants were enrolled in an intensive nutrition-focused intervention emphasizing healthy eating behaviors. However, the observed improvements in readiness for healthy eating—a plausible mediator of the relationship between Food4Moms participation and increased fruit and vegetable intake—provide supportive evidence that the intervention may have contributed, in part, to the dietary changes observed.

It is important to note that the absence of a control group was intentional and guided by ethical considerations raised by community partners, who did not feel it was appropriate to withhold access to fresh produce from pregnant women with low incomes. In addition, available funding constrained the study design to a single-group evaluation. Given that the primary objectives were to codesign the program, assess implementation feasibility, and estimate its potential impact, this approach was appropriate for this phase of research [33] and consistent with other FIM PRx studies [24].

Another limitation is that the Dietary Screener used focused only on fruit and vegetable intake and hence could not be used to assess the overall dietary quality of the participants, including the consumption of ultra-processed foods using the NOVA classification [51]. Furthermore, even though the Fresh Connect card could only be used to purchase fresh produce and redemption rates were quite high, and the Dietary Screening questions were mainly probing for non-ultra-processed foods (see methods section), we could not systematically tease out how much of the fruit and vegetable intake reported in the Dietary Screener could have come from ultra-processed foods. Because high consumption of ultra-processed foods has been consistently associated with a plethora of undesired physical and mental health outcomes [51], future research is needed to understand if and how PRx programs improve not only fresh and minimally processed fruit and vegetable intake but also overall dietary quality, including decreasing the consumption of ultra-processed foods.

It is possible that the participants in our study were more motivated to benefit from our PRx intervention given their relatively lower level of acculturation as proxied by the fact that the great majority chose to respond to surveys in Spanish. Consistent with this hypothesis, acculturation among Latinos has been consistently associated with lower dietary quality [52,53]. Latina Immigrant women consistently describe foods in their countries of origin as fresher and more natural and perceive foods in the United States to be more processed and less flavorful [54]. However, acculturation is not the only driver of food preferences in this population. A recent qualitative study combined with descriptive survey measures that was conducted among immigrant Latina women in the U.S. found that in addition to food availability and cost, family food choices were also shaped by perceived quality, health implications, family dynamics, time constraints, and institutional environments such as schools and day care centers [54]. Acculturation measures did not explain food choices alone as while all participants had relatively low levels of acculturation (proxied by language and the fact that all were born outside the US), dietary acculturation varied widely across individuals. These findings that are consistent with previous acculturation research in the U.S. [55–57] call for further research assessing how the complex web of acculturation, social influences and structural factors may shape food preferences and potentially differential responses to PRx interventions such as Food4Moms. Building on these considerations, we recommend future research based on a randomized controlled trial design to evaluate the effectiveness of Food4Moms more rigorously among persons with different levels of acculturation. We specifically recommend comparing different levels of monthly financial incentives rather than including a no-intervention control group, thereby maintaining ethical integrity while generating evidence to inform scalable program design. Identifying an optimal and cost-effective incentive level will be critical for broader policy translation and program implementation and dissemination.

This study also has several notable strengths. First, it was grounded in a community-engaged, codesign approach, ensuring that the intervention was responsive to participants’ needs and preferences. Program implementation was guided by a quality assurance framework informed by the Program Impact Pathways (PIP) framework and the COM-B model, alongside iterative feedback collected through listening sessions and surveys with participants. This allowed for continuous refinement and adaptation of the program. The use of the COM-B framework was indeed a strength as the association of Food4Moms with produce intake may have been related, at least in part to increasing participants’ capacity (e.g., through culturally tailored skill-building nutrition education sessions), opportunity (by providing user friendly Fresh Connect card, and food retailers that participants were familiar with) and motivation (e.g., through PRx incentives and motivational text messages that can promote the desire to eat healthy during pregnancy to do so.

Second, the study was implemented by the Hispanic Health Council, a trusted community-based organization with more than four decades of experience serving Hispanic populations. Its longstanding presence and established relationships within the community were instrumental in engaging participants, particularly recent immigrants who are often underrepresented in research. The organization’s history of community-based participatory research further strengthened the relevance and acceptability of both the intervention and the evaluation methods.

Third, Food4Moms leveraged an existing, well-established SNAP-Ed program to deliver its interactive nutrition education component. This integration ensured that the intervention was not only evidence-based but also culturally and linguistically appropriate, as well as person-centered in its delivery. As a result, both the program and the research processes were aligned with the cultural context and lived experiences of participants.

Fourth, the partnership with Wholesome Wave provided critical infrastructure for the activation and tracking of Fresh Connect monthly benefits, supporting accurate monitoring of program utilization. Finally, collaboration with an academic partner, the Yale School of Public Health, ensured methodological rigor in study design, data collection, and analysis, strengthening the validity and credibility of the findings.

Moving forward, implementation research is needed to determine how programs like Food4Moms can be effectively scaled and sustained at state and national levels. There is a need to identify the level of financial incentives required to meaningfully improve fruit and vegetable intake while remaining cost-effective and feasible for government agencies and other funders. The optimal duration of benefit provision also warrants further investigation, especially given that infants are recommended to begin complementary feeding around six months of age and could directly benefit from increased household access to healthy foods.

In addition, future studies should evaluate this model across diverse racial and ethnic populations and in larger-scale effectiveness prospective studies with longer follow-up periods. Such research is essential to assess not only dietary outcomes, but also the impacts of programs like Food4Moms on pregnancy, birth, infant feeding, and child health and development outcomes. Generating this evidence will be critical to inform policy decisions and support the broader integration of produce prescription programs into comprehensive maternal and child health strategies.

## Conclusion

Food4Moms is a promising person- and family-centered produce prescription community-based program that may improve readiness for healthy eating, produce intake, and household food security.
